# Emergency Laparotomy and Outcomes in Penetrating Small Bowel Perforation in Unstable Patients

**DOI:** 10.7759/cureus.6022

**Published:** 2019-10-29

**Authors:** Nasim Ahmed, Yen-Hong Kuo, Grace Lepis

**Affiliations:** 1 Surgery, Division of Trauma, Hackensack Meridian Health, Jersey Shore University Medical Center, Neptune, USA; 2 Epidemiology and Public Health, Office of Research Administration, Hackensack Meridian Health Jersey Shore University Medical Center, Neptune, USA; 3 Surgery, Monmouth Medical Center, Long Branch, USA

**Keywords:** penetrating trauma, small bowel injury, hemodynamic instability, timing of laparotomy, mortality and morbidity

## Abstract

Background

The purpose of the study was to evaluate the impact of emergency laparotomy (EL) on outcomes of patients who suffered from small bowel perforations following a penetrating mechanism and presented with initial systolic blood pressure (SBP) <90 mmHg.

Methods

Data from 2012-2014 from the National Trauma Data Bank (NTDB) data set was accessed for this study. All patients who presented with initial SBP <90 mmHg and sustained perforated small bowel injury after a penetrating mechanism and were taken for EL within four hours of the patient’s arrival to the hospital were included in the study. Data were categorized into early group, if the EL was performed within an hour and late group, and if EL was done 1-4 hours of patient arrival to the hospital.

Results

Out of 360, approximately 89% of patients underwent EL within an hour and 11% of patients underwent EL within 1-4 hours of hospital arrival. The median (IQR) time of the late laparotomy was two (2, 3) hours. After propensity matching, there were no significance differences found between the groups regarding in-hospital mortality (11 (26.8%) vs 8 (19.5%), *P* = 0.54), total hospital length of stay (median and IQR 20 (17, 25) vs 15 (11, 20), *P* = 0.117), discharge to home without services (67% vs. 82%, *P* = 0.28), and post-operative complications.

Conclusion

EL in perforated small bowel injury in unstable patients needs to be performed as soon as possible. EL performed within the median of two hours’ time may be acceptable in certain circumstances.

## Introduction

Small bowel injury (SBI) is one of the most common abdominal injuries following a penetrating mechanism and occurs in about 50% of patients, followed by colon and solid organ injuries [1]. The most common penetrating mechanism that causes bowel wall injuries is gunshot wounds (GSW), stab wounds or impaled objects. The distance, trajectory, and caliber of a bullet can lead to not only small bowel injury but can be associated with other intra-abdominal injuries, such as liver, spleen, and vascular injury [2]. These associated abdominal injuries can occur with stab wounds and other impaled objects.

As the number of suicide and homicide incidences in recent years continue to rise, the injury inflicted and the mortality associated with the mechanism will also increase [3-4]. In 2016, the most common mechanism of suicide in the United States was by firearm, and almost 75% of deaths due to homicide resulted from a firearm as well [4-5]. The patient who sustains a penetrating abdominal injury and presents with unstable hemodynamics most commonly suffers from acute blood loss. Concurrent hemorrhage control and replacement of lost blood in an emergent situation are the key steps in reducing the mortality and morbidity [2].

Prior studies have demonstrated that early operative intervention in unstable hemodynamic trauma patients reduces mortality [6]. There is very little controversy regarding the immediate intervention in hemodynamically unstable patients. One of the quality indicators of the American College of Surgeons (ACS) Committee on Trauma is that when hemodynamically unstable patients present with intra-abdominal injuries requiring laparotomy, the laparotomy should be performed within an hour of the patient’s arrival to the hospital. Numerous studies have looked at the timing of operative intervention and outcome following a blunt mechanism in hemodynamically unstable patients; however, there is a paucity of literature related to penetrating bowel injury and timing of operation in unstable patients [7]. Therefore, this study was designed to evaluate the outcomes of patients who underwent emergency laparotomy (EL) using the National Trauma Data Bank (NTDB).

Our hypothesis is EL in perforated small bowel injury in hemodynamically unstable patients reduces overall mortality and morbidity.

## Materials and methods

Data source

The NTDB data set of 2012, 2013 and 2014 was used to conduct this study. The NTDB is currently the largest trauma database worldwide. Nearly 800 medical facilities, ranging from level I to level IV, as well as unranked trauma centers, including both teaching and non-teaching institutions, participate in data sharing with the NTDB, as previously described [7]. The inclusion of patients in the database occurs if the patient’s diagnosis falls into one of the following criteria, as defined by the International Classification of Diseases, Ninth Revision, Clinical Modifier (ICD-9-CM): 800-959.9 excluding the (ICD-9-CM): 905-909.9, 910-924.9 and 930-939.9 [8]. Given that this study was done using a de-identified National database from the ACS that is available to all researchers, this study was exempted from IRB review as per policy and no informed consent was required.

Inclusion and exclusion criteria

All patients who presented with initial SBP <90 mm Hg, who suffered from small bowel perforation following a penetrating mechanism of injury to the abdomen and underwent laparotomy (Procedure code: 54.11) within four hours of hospital admission, were eligible for inclusion in the study. Patient demographics included age, sex, and race, injury severity score (ISS), Glasgow coma scale (GCS), and initial heart rate (HR) were included in the study. Other associated abdominal injuries including liver, spleen, kidney and vascular injuries were also included in the study. Patients were stratified into two groups based on the time of EL; early group consisted of patients who underwent laparotomy within one hour of hospital arrival and the late group consisted of patients who underwent laparotomy after one hour, but not exceeding four hours after hospital arrival.

All patients who sustained abdominal injuries from a blunt mechanism of injury and underwent laparotomy with a small bowel resection were excluded from the study.

Outcomes

The primary outcome of the study was overall in-hospital mortality. The secondary outcome was the length of hospital stay, discharged disposition and in-hospital complication rates including pneumonia, sepsis, urinary tract infections (UTI), deep vein thrombosis (DVT), pulmonary embolism (PE), superficial surgical site infection (SSI), deep SSI, or organ/space SSI.

Statistics

Patient information and outcomes were first summarized using mean with standard deviation (SD), or median with interquartile range (IQR; first quartile to third quartile) for continuous variables, and frequency and percentage for categorical variables. To compare the two groups, the Wilcoxon Rank Sum test was used for continuous variables, and the Chi-square test was used for the categorical variables. The normality of data was tested using the Shapiro-Wilk test.

The propensity score one-to-one matching was performed using the “nearest neighbor” as the matching method to pair a subject who underwent EL within one hour of hospital arrival (early group ) with a subject who underwent EL within 1-4 hours of hospital arrival (late group). The propensity score matching was performed using the R package “MatchIt” [9]. The variables used for calculating the propensity score were age, gender, race, ISS, and GCS. After matching, numerical and graphical diagnostics were used to evaluate the improvement. The patient demographic information and outcomes from the matched subjects were again summarized using mean with standard deviation (SD), or median with IQR for continuous variables, and frequency and percentage for categorical variables as previously described [7,10]. The paired t-test or Wilcoxon Signed Rank test was used to compare the continuous variables between matched groups, depending on the normality of data [7,10]. The McNemar’s test was used to compare the categorical variables between matched groups, if the level of a categorical variable was two. If the level of a categorical variable was more than two, the Stuart-Maxwell test was used [7,10]. The risk difference and 95% confidence intervals were calculated. For the length of total hospital stay, the Kaplan-Meier procedure was used to estimate the median time, and the standard error was estimated using Greenwood’s formula. The Kaplan-Meier curves were generated. The log-rank test was used to compare the time (Kaplan-Meier curves) between groups. The two-sided *p*-value was reported for each test. A *p*-value of < 0.05 was considered an indication of statistical significance. Statistical analysis was performed using the R language [11].

## Results

A total of 360 patients fulfilled the inclusion criteria of the study. Out of those patients, 319 (88.6%) patients underwent laparotomy early and only 41 (11.4%) patients underwent laparotomy within 1-4 hours, median (IQR) time was two (2, 3) hours of hospital arrival. In the late group, 30/41 patients underwent EL within two hours, only 6/41 and 5/41 underwent laparotomy within three and four hours, respectively. Overall, the median (IQR) age of the patients was 30 (23, 43) years, predominantly males (90.56%), and 53.89% of patients were African Americans. A majority, 85.83%, of patients sustained firearm injuries and 14.17% of patients suffered from stab wound injuries. Approximately 84% of the stab wounds and 89% of GSW underwent laparotomy within an hour of hospital arrival, *P* =0.29. Liver injury was the most common associated abdominal injury, found in 18.6% of patients, followed by kidney injury in 14.4% of patients and splenic injury in 10% of patients.

On univariate analysis, there were differences in the patient characteristics amongst groups regarding SBP median (IQR) was (74 mm Hg (60-81) vs 80 mm Hg (76-85), *P *= 0.002)), other venous injury (10% vs. 0%, *P *= 0.036), and the anatomical location where the injury occurred (*P *= 0.006; Table 1).

**Table 1:** Patient’s characteristics before propensity matching ISS, injury severity score; GCS, Glasgow coma scale; SBP, systolic blood pressure; IVC, inferior vena cava; ACS, American College of Surgeons

	Early Group (n = 319)	Late Group (n = 41)	P-Value
Age			0.444
Median (Q1-Q3)	30 (23-43)	31 (25-40)	
Gender			1.0
Female	30 (9.4)	4 (9.8)	
Male	289 (90.6)	37 (90.2)	
Race			0.63
Black	172 (53.9)	22 (53.7)	
Other	53 (16.6)	9 (22)	
White	94 (29.5)	10 (24.4)	
ISS			0.356
Median (Q1-Q3)	18 (14-26)	19 (16-27)	
GCS			0.059
Median (Q1-Q3)	14 (3-15)	15 (12-15)	
SBP			0.002
Median (Q1-Q3)	74 (60-81)	80 (76-85)	
Pulse			0.977
Median (Q1-Q3)	104 (83-128)	107 (91-120)	
Spleen injury, n (%)	33 (10.3)	3 (7.3)	0.782
Liver injury, n (%)	57 (17.9)	10 (24.4)	0.426
Kidney injury, n (%)	48 (15)	4 (9.8)	0.502
Other vein injury, n (%)	32 (10)	0 (0)	0.036
IVC injury, n (%)	33 (10.3)	2 (4.9)	0.401
Iliac vein injury, n (%)	20 (6.3)	5 (12.2)	0.184
Common iliac vein, n (%)	34 (10.7)	1 (2.4)	0.155
Other arterial injury, n (%)	33 (10.3)	3 (7.3)	0.782
Iliac artery injury, n (%)	48 (15)	4 (9.8)	0.502
Celiac artery injury, n (%)	3 (0.9)	0 (0)	1.0
Aortic injury, n (%)	18 (5.6)	0 (0)	0.243
Teaching status, n (%)			0.707
Community	71 (22.3)	11 (26.8)	
Non-teaching	15 (4.7)	2 (4.9)	
University	233 (73)	28 (68.3)	
Bed size, n (%)			0.582
≤200	9 (2.8)	2 (4.9)	
201-400	75 (23.5)	9 (22)	
401-600	96 (30.1)	15 (36.6)	
>600	139 (43.6)	15 (36.6)	
ACS trauma level, n (%)			0.148
Unavailable	1 (0.3)	1 (2.4)	
I	141 (44.2)	13 (31.7)	
II	44 (13.8)	7 (17.1)	
Not Applicable	133 (41.7)	20 (48.8)	
Region			0.006
Midwest	67 (21.6)	15 (42.9)	
Northeast	65 (21)	10 (28.6)	
South	137 (44.2)	8 (22.9)	
West	41 (13.2)	2 (5.7)	

In order to remove selection bias, propensity score matching was performed between the groups. Propensity score matching is a pairing technique that is based on the subject’s propensity (or likelihood) to be exposed to something or have a certain trait based on the existing characteristics. After propensity score matching and pair-matched analysis between the two groups, there were no significant differences found in the above variable except ISS. The median and IQR ISS was lower in the early group compared to the late group (18 {10, 25} vs. 19 {16, 27}, *P *< 0.001), respectively (Table 2).

**Table 2:** Patient characteristics after propensity matching ISS, injury severity score; GCS, Glasgow coma scale; SBP, systolic blood pressure; IVC, inferior vena cava; ACS, American College of Surgeons

	Early Group (n=41)	Late Group (n=41)	P-Value
Age			0.82
Median (Q1-Q3)	32 (22-45)	31 (25-40)	
Gender, n (%)			1.0
Female	5 (12.2)	4 (9.8)	
Male	36 (87.8)	37 (90.2)	
Race, n (%)			0.497
Black	20 (48.8)	22 (53.7)	
Other	8 (19.5)	9 (22)	
White	13 (31.7)	10 (24.4)	
ISS			0.0004
Median (Q1-Q3)	18 (10-25)	19 (16-27	
GCS			0.723
Median (Q1-Q3)	15 (13-15)	15 (12-15)	
SBP			0.285
Median (Q1-Q3)	80 (68-82)	80 (76-85)	
Pulse			<0.0001
Median (Q1-Q3)	110 (81-129)	107 (91-120)	
Spleen injury, n (%)	4 (9.8)	3 (7.3)	1.0
Liver injury, n (%)	6 (14.6)	10 (24.4)	0.386
Kidney injury, n (%)	7 (17.1)	4 (9.8)	0.546
Other vein injury, n (%)	6 (14.6)	0 (0)	NA
IVC injury, n (%)	6 (14.6)	2 (4.9)	0.289
Iliac vein injury, n (%)	2 (4.9)	5 (12.2)	0.45
Common iliac vein, n (%)	3 (7.3)	1 (2.4)	0.617
Other arterial injury, n (%)	5 (12.2)	3 (7.3)	0.724
Iliac artery injury, n (%)	3 (7.3)	4 (9.8)	1.0
Celiac artery injury, n (%)	1 (2.4)	0 (0)	NA
Aortic injury, n (%)	2 (4.9)	0 (0)	NA
Teaching status, n (%)			0.714
Community	11 (26.8)	11 (26.8)	
Non-teaching	4 (9.8)	2 (4.9)	
University	26 (63.4)	28 (68.3)	
Bed size, n (%)			0.765
≤200	2 (4.9)	2 (4.9)	
201-400	12 (29.3)	9 (22)	
401-600	11 (26.8)	15 (36.6)	
>600	16 (39)	15 (36.6)	
ACS trauma level, n (%)			0.875
Unavailable	1 (2.4)	1 (2.4)	
I	10 (24.4)	13 (31.7)	
II	7 (17.1)	7 (17.1)	
Not Applicable	23 (56.1)	20 (48.8)	
Region, n (%)			0.121
Midwest	8 (20.5)	15 (42.9)	
Northeast	7 (17.9)	10 (28.6)	
South	19 (48.7)	8 (22.9)	
West	5 (12.8)	2 (5.7)	

The overall in-hospital mortality after matching between the early and late groups was (11 {26.8%} vs 8 {19.5%}, *P *= 0.54) respectively, and the absolute risk difference was 0.073 (-0.108, 0.255). No significant difference was found regarding hospital length of stay, median (IQR; 20 (17, 25) vs 15 (11, 20), *P* = 0.117) between the two groups. Besides, no differences were found regarding intensive care unit (ICU) days (6 (3, 10) vs 4.5 (2, 12.5), *P *= 0.88), and days on ventilator (4 (2, 6) vs. 5 (2, 8.5), *P *= 0.68) between the early and late groups, respectively (Table 3).

**Table 3:** In-hospital mortality and length of stay after matching ICU, intensive care unit

	Early Group (n = 41)	Late Group (n = 41)	P-Value
Died	11 (26.8)	8 (19.5)	0.546
Total hospital length of stay; Median (95% CI; Kaplan-Meier procedure)	20 (17, 25)	15 (11, 20)	0.117
ICU days	n = 31	n = 32	0.88
Median (Q1-Q3)	6 (3, 10)	4.5 (2, 12.5)	
Vent days	n = 30	n = 31	0.68
Median (Q1-Q3)	4 (2, 6)	5 (2, 8.5)	

For patients who survived until hospital discharge, no significant differences were found on discharge disposition (*P* = 0.208; Table 4).

**Table 4:** Discharge disposition of patients who survived to discharge

Discharge disposition	Early Group (n = 30)	Late Group (n = 33)	P-Value
			0.208
Another Hospital	4 (13.3)	0 (0)	
Home: Healthcare	2 (6.7)	1 (3)	
Home: No Services	20 (66.7)	27 (81.8)	
Intermediate care	0 (0)	1 (3)	
Long-term care	3 (10)	2 (6.1)	
Skilled nursing care	1 (3.3)	2 (6.1)	

When assessed for postoperative complications in pair-matched analysis, no significant differences were found between the groups regarding the incidences of DVT, PE, acute kidney injury, UTI, pneumonia, severe sepsis, and surgical-site infection (Table 5).

**Table 5:** Pair-matched comparison of complications between the groups ICU, intensive care unit; OR, odds ratio

Complications	Early Group (n=41)	Late Group (n=41)	P-Value
Acute kidney injury, n (%)	2 (4.9)	2 (4.9)	1
Deep vein thrombosis, n (%)	1 (2.4)	3 (7.3)	0.617
Pulmonary embolism, n (%)	1 (2.4)	1 (2.4)	1
Superficial surgical site infection, n (%)	2 (4.9)	1 (2.4)	1
Deep surgical site infection, n (%)	1 (2.4)	1 (2.4)	1
Organ/space surgical site infection, n (%)	2 (4.9)	4 (9.8)	0.683
Pneumonia, n (%)	4 (9.8)	4 (9.8)	1
Urinary tract infection, n (%)	6 (14.6)	1 (2.4)	0.131
Severe sepsis, n (%)	2 (4.9)	2 (4.9)	1
Unplanned return to the ICU, n (%)	1 (2.4)	2 (4.9)	1
Unplanned return to the OR, n (%)	3 (7.3)	2 (4.9)	1

Further analysis of the late group showed that the majority of the patients (32/41) underwent EL within two hours and only 11/41 patients whose laparotomy time was 3-4 hours of hospital arrival. None of these 11 patients suffered from any abdominal solid organs (liver, spleen, and kidney) injuries. Only three of these 11 patients in the late group were found to have iliac artery injuries.

## Discussion

Our study evaluated all patients from NTDB who inflicted with penetrating mechanism, sustained with small bowel injury and presented with unstable hemodynamics. The majority of these patients (89%) underwent immediate laparotomy as recommended by ACS. A small fraction of patients (11%) underwent EL late. However, even in a late group, the median time of EL was two hours after hospital arrival. Our study showed that patients not only suffered from perforation of small bowel but also sustained other solid abdominal organ and vascular injuries as reported by other [12]. The most common solid organ injury found was liver, followed by kidney and spleen. The common vascular injuries were iliac vein (17% of cases), iliac artery (14% of cases) and IVC injury (10% of cases). After pair-matched analysis when all patients’ characteristics including other associated injuries were compared. We found no statistically significant difference except in ISS. There was a one-point difference in median ISS between the groups and higher ISS was seen in the late laparotomy group. 

There were no statistically significant differences found in overall in-hospital mortality, total hospital length of stay, ICU days and ventilator days between the two groups. No significant differences were found between the two groups regarding postoperative complications and discharge to home without services as well. The most probable reason for no differences in outcomes in the early vs late laparotomy group was a majority (97%) of patients underwent EL within the two hours of patient’s arrival to the hospital. Glance and colleagues reported the trauma quality indicators and its outcome evaluating the Pennsylvania Trauma Center's data found the compliance rate with the two hours window of EL were in 81 % of cases [13]. In their analysis delayed in laparotomy more than two hours was associated with a higher risk of death or major complications, odds ratio 1.42; 95% CI, (1.19-1.69).

Penetrating injury as a result of gunshots to the abdomen is usually associated with hollow viscus injury and requires immediate surgical exploration, particularly when the patient is hemodynamically unstable. The location of the injury, velocity, and trajectory of the projectile often determines the organ injury and the severity of injury [14]. Knife wounds, on the other hand, are associated with a lower incidence of intra-abdominal injury, and hence, a proper workup requires clinical judgment and experience. The prognosis of patients with penetrating abdominal trauma is variable and depends on the extent of injury and time of presentation to the emergency department [15]. Peritoneal violation occurs in 50% to 70% of patients with abdominal stab wounds, but only half of those patients with peritoneal violation sustain an intra-abdominal injury requiring operative intervention [16]. Thus, only 25% to 33% of patients with abdominal stab wounds require laparotomy.

When a patient arrives in the trauma bay with a penetrating abdominal injury, they should be immediately assessed for signs of generalized peritonitis, hemodynamic instability, impalement, and evisceration. Typically, if patients experience any signs or symptoms, as mentioned above, they are resuscitated and immediately transported to the operating room for an emergent laparotomy. Early laparotomy for perforated viscus with massive abdominal contamination, hemorrhage, multi-organ injury, associated head injury, or coagulopathy resulted in survival benefit.

Prior studies have reported some success in selective non-operative management (NOM) in penetrating abdominal trauma in hemodynamically stable patients in a setting of close monitoring. However, the failure of NOM and delay in operative intervention did not carry any additional risk of the increase in mortality [17]. Velmahos et al conducted a study of 1,856 patients with abdominal gunshot wounds. Initially, 792 (42%) of those patients were managed non-operatively, however, 80 (4%) of these patients underwent delayed surgery. The delay in laparotomy was between three and 48 hours after the patient’s arrival at the hospital. No mortality was reported in the delayed cases, only 5 out of 80 patients developed complications. There was a longer hospital length of stay in patients with failure of no operative management. Approximately 14% among operated patients (or 9% of total patients) patients underwent a non-therapeutic laparotomy [18].

Leppäniemi et al. performed a prospective randomized trial looking at safety and cost-effectiveness of selective, non-operative management compared to mandatory laparotomy in patients with abdominal stab wounds not requiring immediate laparotomy for hemodynamic instability, peritonitis, or evisceration of abdominal [12]. Fifty-three percent of patients were assigned to mandatory laparotomy and 47% non-operative management and compared for morbidity, length of stay, and hospital cost. The study found that there was no significant early mortality. The morbidity rate was found to be 19% following mandatory laparotomy but only 8% after observation (*p*-value = 0.26), which is not statistically significant. This study concluded that selective non-operative management of penetrating abdominal stab wounds, although resulting in delayed laparotomy in some cases, however, did not impact the outcomes. The same group performed a follow-up retrospective study of 209 patients [19]. If the patients showed signs of unstable hemodynamics or generalized peritonitis within three hours of initial presentation, the patients were immediately taken to the operating room and performed a laparotomy. Six patients with hemodynamic instability found to have no significant abdominal organ injury. Five out of six patients had arterial bleeding from the abdominal wall or from the liver injury that was stopped when laparotomy was performed.

Similarly, Demetriades and colleagues evaluated 651 patients with an anterior stab wound, 53% of patients underwent immediate laparotomy for signs of acute generalized peritonitis, and 306 out of 651 patients were observed [20]. Approximately 6% of patients in the observed group presented with signs of shock. None of these patients required an operation. The definition of immediate operation does not give a time frame, however, delayed laparotomy in their study ranges from four hours to five days. Delay in laparotomy did not cause any death and only two patients developed wound sepsis.

Contrary to the above studies, the current study showed the all patients needed to have laparotomy as soon as possible due to instability in hemodynamics. Since shock on admission, a harbinger of significant blood loss and abdominal organ injury, immediate laparotomy is recommended [21]. A majority of our patients did undergo laparotomy rather quickly. However, a fraction of patients had a little delay in their laparotomies. The reasons for the delay were not provided and in most cases, EL was performed within two hours of patient arrival to the hospital. The delay in laparotomy did not lead to an increase in mortality, morbidity or hospital length stay.

Limitations

The study was performed using the largest trauma data repository worldwide; however, it carries the same inherent limitation as any other data repository. This is a retrospective study, and therefore, the event has already taken place. Although the propensity score matching analysis was used to remove some of the selection bias, the analysis does not consider the unmeasured characteristics. Furthermore, this study was performed in a selective group of patients, and the sample size was considerably small, and therefore, the interpretation of results should be taken with caution. NTDB also does not provide detailed information on the patient’s response to the immediate management, and therefore, it is difficult to determine the cause of delayed laparotomy in some cases.

## Conclusions

Patients who presented with perforated small bowel with a penetrating mechanism of injury and hypotension also found to suffer from other intra-abdominal solid organ injuries and vascular injuries. In these patients, the resuscitation should be started immediately and emergency laparotomy needs to be performed as soon as possible. Performing the EL within the median time of two hours of the patient’s arrival did not adversely impact the outcomes. 
